# The Effect of Comprehensive and Integrative Medical Services on Patients with Degenerative Lumbar Spinal Stenosis: A Randomized Controlled Study

**DOI:** 10.3390/medicina62010225

**Published:** 2026-01-21

**Authors:** Sang Bong Ko, Sang Gyu Kwak, Hee Chan Kim

**Affiliations:** 1 Department of Orthopaedic Surgery, School of Medicine, Daegu Catholic University, Daegu Catholic University Hospital, Daegu 42472, Republic of Korea; 2 Department of Medical Statistics, School of Medicine, Daegu Catholic University, Daegu 42472, Republic of Korea; sgkwak@cu.ac.kr; 3 Department of Orthopaedic Surgery, Armed Forces Daegu Hospital, Gyeongsan 425-95, Republic of Korea; glck7946@naver.com

**Keywords:** spinal stenosis, selective nerve root block, acupuncture, integrative medicine, multimodal therapy, neuropathic pain

## Abstract

*Background and Objectives*: Degenerative lumbar spinal stenosis (DLSS) frequently manifests as lower leg radiating pain (LLRP), requiring selective nerve root block (SNRB). Comprehensive and Integrative Medical Services (CIMS)—a multimodal program consisting of acupuncture, cupping, and manual therapy—have been increasingly incorporated into clinical practice in Korea. However, randomized evidence remains limited. This study evaluated the efficacy and safety of adjunctive CIMS in patients with DLSS presenting neuropathic LLRP requiring SNRB. *Materials and Methods*: In a single-center, parallel-group, assessor-blinded randomized controlled trial (CRIS KCT0006036), adults with DLSS (LANSS > 7; VAS > 5) were randomized 1:1 to experimental or control groups (*n* = 77; experimental 38, control 39). All participants received SNRB plus pharmacotherapy (limaprost, pregabalin). The experimental group additionally received CIMS, delivered eight times over 4 weeks. The primary outcome was pain intensity (VAS) at baseline and weeks 4, 8, and 12. Secondary outcomes included SF-36, ODI, and RMDQ at baseline and weeks 4, 8, and 12. Repeated-measures two-factor ANOVA assessed the main effects and time × group interaction. *Results*: Mean VAS (experimental vs. control) was 4.73 ± 1.67 vs. 4.70 ± 1.95 at baseline; 3.74 ± 1.68 vs. 4.66 ± 1.60 at week 4; 3.93 ± 2.03 vs. 4.79 ± 1.55 at week 8; and 3.98 ± 1.98 vs. 4.98 ± 1.68 at week 12. The significant time × group interaction was identified (*p* = 0.040), indicating a greater pain reduction with CIMS. No significant time × group interactions were observed across SF-36 domains. Adherence to CIMS modalities was high, and no unexpected adverse events occurred. *Conclusions*: In DLSS patients receiving SNRB and pharmacotherapy, adjunctive CIMS resulted in greater pain reduction over 12 weeks compared with standard care alone, without introducing new safety concerns. These findings support the clinical utility of CIMS as an effective adjunctive treatment option for DLSS.

## 1. Introduction

Degenerative lumbar spinal stenosis (DLSS) is a prevalent degenerative spinal disorder characterized by narrowing of the spinal canal and subsequent compression of the spinal roots. This compression commonly leads to neurogenic intermittent claudication (NIC), lower leg radiating pain (LLRP), sensory disturbance, motor weakness, and low back pain (LBP) [1]. Both LLRP and referred buttock pain are typically described as stabbing, dull, burning, electric shock-like, numb, or paresthesia-like sensations, and are reported in the majority of patients. Conservative treatment is generally attempted initially; however, when symptoms worsen, functional impairment progresses, or neurological deficits emerge, surgical intervention may be considered due to its substantial impact on quality of life [2].

Conservative treatment options for DLSS include pharmacotherapy, physical therapy, therapeutic exercise, and injection therapies as epidural steroid injections and selective nerve root block (SNRB). In Korea, both western and traditional oriental medical practices coexist. Among oriental modalities, previous studies have reported that acupuncture can reduce pain intensity and improve functional outcomes in patients with lumbar spinal stenosis; however, the magnitude of benefit varies across studies, and limitations such as small sample sizes and heterogeneity of protocols have been noted [3,4,5,6]. Manual therapy—including joint mobilization or correction, soft tissue mobilization, neural mobilization, and stabilization exercises—has also been shown to reduce pain and improve physical function in DLSS [7]. While these studies support the effectiveness of individual non-surgical modalities, evidence synthesizing combined integrative approaches for DLSS remains limited.

Since 2017, the Ministry of Health and Welfare of Korea has promoted a collaborative care model through the “Western-Oriental Medicine Cooperation Pilot Project”, which evolved into a comprehensive integrative medical service (CIMS) model. CIMS combines mechanism-targeted analgesic interventions (e.g., acupuncture), functional restoration strategies (manual therapy and exercise), and microcirculatory enhancement (cupping) [8]. Although multimodal integrative approaches conceptually align with the multifactorial pathophysiology of DLSS and may yield synergistic therapeutic effects, systematic reviews or meta-analyses evaluating predefined combined integrative treatment protocols for DLSS are currently lacking, largely due to heterogeneity in intervention components and limited randomized evidence. From a mechanistic perspective, acupuncture primarily targets nociceptive modulation and neuroinflammatory pathways, manual therapy focuses on biomechanical correction and functional restoration, and cupping therapy may enhance local microcirculation and muscle relaxation. The integration of these modalities may therefore provide complementary effects on both pain generation and functional impairment in DLSS.

In a previous pilot randomized controlled study published in 2023, involving a small sample of patients with DLSS, a 6-week, 12-session CIMS program demonstrated greater improvements in pain intensity (VAS) compared with conventional care alone, although changes in SF-36 domains were not statistically significant [9].

Building upon the methodology and preliminary results of that pilot study, we designed the present assessor-blinded randomized controlled study with a larger sample size and a clinically optimized treatment schedule. This study compared a CIMS-augmented treatment group—receiving acupuncture, cupping therapy, and manual therapy eight times over 4 weeks—with a control group receiving standard care only (limaprost, pregabalin, and SNRB). Limaprost, pregabalin, and SNRB are commonly used components of standard conservative management for DLSS in Korea, particularly in patients presenting with neuropathic radiating pain. This study aimed to evaluate the effects of adjunctive CIMS on pain reduction, functional improvement, overall health status, and safety in patients with DLSS.

## 2. Materials and Methods

### 2.1. Study Design and Patient Selection

This study was a single-center, parallel-group, randomized, assessor-blinded controlled trial (registered at https://cris.nih.go.kr, accessed on 16 January 2026; KCT 0006036; date of trial registration: 29 March 2021). The study protocol was approved by the Institutional Review Board of Daegu Catholic University Medical Center (approval number: 2022-07-009; date of approval: 28 September 2022). Written informed consent was obtained from all participants prior to enrollment.

Eligible patients were adults diagnosed with degenerative lumbar spinal stenosis and presenting with radiating pain requiring selective nerve root block (SNRB). Neuropathic characteristics of radiating pain were confirmed using the Leeds Assessment of Neuropathic Symptoms and Signs (LANSS), and individuals with a LANSS score > 7 and a visual analog scale (VAS) score > 5 were included. Inclusion and exclusion criteria are detailed in Table 1.

The study was conducted and reported in accordance with the CONSORT 2025 guidelines, with the completed checklist and participant flow diagram provided in the Supplementary Materials [10].

Based on prior results [9], the primary endpoint was VAS. Sample size calculations (α = 0.05, power = 0.80, 10% dropout rate) indicated 40 participants per group (total *n* = 80). Details are shown in Table 2 and Table 3.

### 2.2. Treatment Procedures

All participants received SNRB and pharmacotherapy with limaprost and pregabalin. In addition to standard care, the experimental group received CIMS consisting of acupuncture, cupping therapy, and a manual therapy-based massage. CIMS sessions were administered 8 times over a 4-week period.

#### CIMS Component and Protocol

1. Acupuncture:

Acupuncture treatment followed clinical practice guidelines in oriental medicine for DLSS and prior studies evaluating acupuncture following SNRB [11]. Proximal acupuncture points included Shenshu (BL23), Qihai-shu (BL24), Dachang-shu (BL25), and Huatuo Jiaji (EX-B2). Given the predominance of LLRP in DLSS, distal points included Huantiao (GB30), Weizhong (BL40), and Lunlun (BL60). Disposable sterile filiform needles (Dongbang Acupuncture Inc., Boryung, Seoul, Republic of Korea) (0.25 × 40 mm and 0.30 × 60 mm) were used, totaling 17 needles per session. Needle insertion was performed with manual stimulation to achieve deqi sensation, needles were retained for 25 ± 5 min. Electroacupuncture was applied at BL23, BL25, and EX-B2 with a frequency of 4 Hz stimulation for 25 ± 5 min, with current intensity adjusted according to patient tolerance. Needle placement and stimulation parameters were standardized across sessions and verified by experienced practitioners to ensure treatment consistency.

2. Cupping Therapy:

Tenderness was identified through palpation of paraspinal and back-shu regions, and negative pressure was applied at the standardized level using an electric suction device (Leaders Meditech multicare, Leaders Meditech Co., Ltd., Yongin, Republic of Korea). Negative pressure was maintained for 5 min. Cupping therapy is intended to promote microcirculation, lymphatic flow, immune activity, and relief of muscle tension-related pain. Previous meta-analyses have demonstrated its clinical benefits, particularly when combined with acupuncture, in conditions such as herpes zoster, facial palsy, and cervical spondylosis [12].

3. Manual therapy-based intervention:

The manual therapy program consisted of 30 min sessions conducted 8 times over 4 weeks. All manual therapy sessions were delivered by therapists with formal training in musculoskeletal manual therapy, following a standardized protocol to ensure consistency across participants. The protocol included palpatory assessment to identify myofascial restrictions, mobilization techniques targeting paraspinal muscles and associated ligaments, myofascial release, therapeutic massage, and stretching exercises, all aimed at alleviating stenosis-related pain and improving functional mobility of the paraspinal muscles and adjacent joints.

4. Point selection summary:
  - Acupuncture points: BL23, BL24, BL25, EX-B2 (L2–L5), GB30, BL40, and BL60.
  - Cupping points: Tenderness-related back-shu points (the regions of maximal pain).
  - Manual therapy: 30 min per session, 8 sessions total.
  - Treatment frequency: 8 sessions over 4 weeks.

### 2.3. Randomization and Blinding

Participants were randomized at a 1:1 ratio to either the experimental or control group. Due to the nature of the interventions, participant blinding was not feasible; however, outcome assessors remained blinded to group allocation throughout the study period. The randomization sequence was generated using a computer-based random number algorithm by an independent statistician and implemented using sequentially numbered codes to ensure allocation concealment. Participants were assigned sequential randomization codes (R001, R002, R003, …) upon enrollment. This study employed single blinding, wherein the outcome assessors were blinded to group allocation throughout data collection and evaluation.

### 2.4. Outcome Measures

At screening visit, demographic data including initials, sex, age, height, weight, comorbidities, and LANSS were recorded. The primary outcome was pain intensity assessed by the VAS at baseline, week 4, week 8, and week 12. Physical function and health status were assessed using the 36-item Short-Form Health Survey (SF-36), and spine-related disability was evaluated using the Oswestry Disability Index (ODI) and Roland–Morris Disability Questionnaires (RMDQ) at baseline and at week 4, 8, and 12 in both groups. Compliance in the experimental group was evaluated at week 4. For each modality (acupuncture, cupping, and manual therapy-based intervention), adherence (%) was calculated as (number of sessions attended/8) × 100. Participants with less than 70% adherence were classified as dropouts.

### 2.5. Statistical Analysis

All statistical analyses were performed in accordance with national clinical trial statistical guidance by the Korea Food and Drug Administration (KFDA, 2000). Analyses were conducted using IBM SPSS Win. Ver. 19.0 (IBM Corp., Armonk, NY, USA). A two-sided α level of 0.05 was considered statistically significant. Normality assumptions were assessed prior to analysis, and parametric or non-parametric tests were selected accordingly.

Descriptive statistics were used to summarize baseline demographic and clinical characteristics. Continuous variables were presented as means ± standard deviations and categorical variables as frequencies and percentages. Between-group homogeneity at baseline was assessed using independent two-sample t-tests for normally distributed continuous variables, Mann–Whitney U tests for non-normally distributed variables, and chi-square tests for categorical variables.

For efficacy outcomes (VAS, SF-36, ODI, and RMDQ), repeated-measures two-factor ANOVA was used to analyze changes over time, with factors for time, group, and time × group interactions. Assumptions underlying repeated-measures ANOVA, including normality and sphericity, were evaluated prior to analysis. When significant interactions were observed, planned contrast analyses were performed to identify specific time points showing group differences. Efficacy analyses were performed using a modified intention-to-treat (mITT) population, including all randomized participants who completed at least one post-baseline outcome assessment. Missing data were not imputed, and analyses were conducted using observed data only. Safety analyses included all participants who underwent at least one CIMS session. Adverse events (AEs) were documented and compared between groups using chi-square tests. The proportion of participants experiencing ≥ 1 AE was also evaluated using chi-square tests.

In addition to *p*-values, standardized effect sizes were calculated to quantify the magnitude of treatment effects. Cohen’s d was used for between-group differences in change scores.

### 2.6. Safety Assessment

All adverse events (AEs) were monitored throughout the study period at each visit. AEs were defined as any unfavorable or unintended medical occurrence temporally associated with the intervention, regardless of causal relationship. The severity of AEs was assessed based on clinical judgment and classified as mild, moderate, or severe. Appropriate medical management was provided as needed, and serious adverse events were to be reported to the institutional review board.

## 3. Results

### 3.1. Participant Flow and Baseline Characteristics

A total of 77 participants met inclusion and exclusion criteria and were randomized into either the experimental group (*n* = 38; 11 men, 27 women) or the control group (*n* = 39; 14 men, 25 women). Mean age did not differ significantly between groups (experimental 69.74 ± 6.29 years; control 69.28 ± 7.32 years; *p* = 0.771). All participants exhibited neuropathic radiating pain, with LANSS scores > 7 in both groups. The distribution of comorbid medical conditions differed between groups (experimental *n* = 4; control *n* = 0) (Table 4).

### 3.2. Compliance in the Experimental Group

For the manual therapy-based intervention, 31 of 38 participants achieved 100% adherence; one participant discontinued after seven sessions due to symptomatic improvement. For acupuncture, 32 of 38 participants achieved 100% adherence. In each group, 6 participants discontinued the study. Reasons for discontinuation included symptomatic improvement, withdrawal of consent, and loss to follow-up; no discontinuations were related to serious adverse events. Overall adherence rates were high, with the majority of participants completing all scheduled sessions, suggesting adequate treatment exposure for efficacy evaluation.

### 3.3. Safety Outcomes

No adverse events were reported in either group during the study period. In particular, no serious adverse events related to acupuncture, cupping therapy, manual therapy, pharmacotherapy, or SNRB were observed.

### 3.4. Changes in VAS, RMDQ, ODI

Mean VAS scores in the experimental group decreased from 4.73 ± 1.67 at baseline to 3.74 ± 1.68 at week 4, 3.93 ± 2.03 at week 8, and 3.98 ± 1.98 at week 12. In the control group, the corresponding scores were 4.70 ± 1.95, 4.66 ± 1.60, 4.79 ± 1.55, and 4.98 ± 1.68, respectively. Repeated-measures two-factor ANOVA revealed a significant time × group interaction for VAS (*p* = 0.040), indicating greater improvement in the experimental group. For disability outcomes, ODI demonstrated a significant time × group interaction (*p* = 0.010), consistent with superior functional improvement in the experimental group. However, no significant time × group interaction was observed for RMDQ (*p* = 0.418) (Table 5).

### 3.5. Changes in SF-36

No statistically significant time × group interactions were observed across any SF-36 domains (all *p* > 0.05), indicating that the 12-week intervention period may have been insufficient to influence multidimensional quality-of-life measures (Table 6).

## 4. Discussion

Degenerative lumbar spinal stenosis (DLSS) is a common degenerative spinal disorder that often leads to chronic pain, functional disability, and reduced quality of life. In a previous study, approximately half of patients treated conservatively experienced symptomatic improvement over 4 years, whereas a notable proportion reported persistent or worsening leg or back pain [13]. Consequently, conservative management remains the initial approach when neurological deficits are not severe, with surgical intervention reserved for refractory cases or significant functional impairment.

Conservative treatment encompasses patient education and activity modification, pharmacological therapies (e.g., anti-inflammatory agents, pregabalin, muscle relaxants, limaprost), injection therapies (e.g., epidural corticosteroid injections and selective nerve root blocks), spinal manipulation, bracing, and physical therapy and exercise [14,15]. When conservative management fails, surgical decompression—with or without fusion—may be considered.

Interest in collaborative integrative medical services (CIMS) as an adjunct to standard care has grown in Korea. Since 2017, a national ministry-led pilot program (Phase 3) has supported the implementation of CIMS, integrating acupuncture, cupping, manual therapy, and related modalities with conventional western medical care [9]. Previous real-world evidence have supported the clinical utility of integrative treatment programs [16]. Our previous pilot study also demonstrated that 12 sessions of CIMS improved pain more effectively than conventional care alone [8].

In the present trial, we expanded the sample size and employed a shorter, clinically practical treatment regimen of eight sessions over 4 weeks. Despite the reduced treatment intensity, we again observed significantly greater improvement in VAS and ODI in the CIMS group compared with standard care alone. Beyond statistical significance, the observed improvements also appear clinically meaningful. Although the within-group reduction in VAS in the experimental group did not reach 1.5–2.0 points, the experimental group maintained pain improvement over 12 weeks whereas the control group showed slight worsening, resulting in an approximately 1.0 point between-group difference at week 12. ODI improvement surpassed the suggested MCID of 10–12 points for lumbar spine disorders, indicating the benefits of adjunctive CIMS are likely to be perceptible and relevant to patients. These findings strengthen evidence supporting the role of CIMS as a beneficial adjunctive treatment strategy for DLSS.

It is notable that SF-36 outcomes did not differ significantly between groups, consistent with our prior pilot study. Quality-of-life measures such as SF-36 reflect multidimensional constructs beyond pain and physical function. In chronic DLSS, improvements in pain may not immediately translate into measurable quality-of-life changes, particularly within a 12-week timeframe. Quality-of-life domains may require longer treatment duration, more intensive multidisciplinary psychosocial interventions. The persistence of this pattern across studies suggests that pain reduction alone may not suffice to alter complex, multidimensional quality-of-life indicators.

Although no additional statistical adjustment was performed due to the small number of affected participants, this imbalance is acknowledged as a potential confounder and should be considered when interpreting functional outcomes. Although formal effect size estimates were not calculated, the observed between-group differences exceeded established MCID thresholds, supporting the clinical relevance of the findings.

A multicenter Korean study involving 387 inpatients with lumbar spinal stenosis who received integrative oriental medicine for a mean of 3 weeks reported clinically meaningful improvements in pain and function, as well as a high rate of surgery avoidance [17]. These findings are consistent with our results and suggest that CIMS may contribute not only to short-term symptom relief but also to long-term functional recovery.

Mechanistically, CIMS may exert complementary effects through multiple pathways [18]. Acupuncture has been shown to modulate nociceptive ion channels and neuroinflammatory responses [19,20]. Manual therapy and exercise may enhance muscle activation and central pain inhibition, leading to improved short-term pain and disability [21,22,23,24]. Cupping therapy can improve microcirculation and lymphatic flow, thereby relieving pain related to muscle tension [25,26]. The combination of these modalities may produce synergistic effects that address both the nociceptive and functional components of DLSS. The high adherence rate and absence of significant adverse events in this study further support CIMS as a feasible adjunctive intervention.

A key distinction between the present and pilot studies is the treatment intensity and duration. Whereas the pilot utilized 12 sessions across 6 weeks, this study implemented a condensed, pragmatic eight-session, 4-week protocol. Comparable clinical trends between the two trials suggest that shorter CIMS programs may still provide meaningful benefit and may be more feasible for routine clinical practice.

This study has several limitations. First, the single-center design may limit the external validity of the findings. Second, the 12-week follow-up period was insufficient to evaluate long-term outcomes such as symptom recurrence or delayed functional improvement. Third, single-blind design could introduce observational bias, given the impossibility of blinding participants to manual or acupuncture therapies. Fourth, the individual contributions of acupuncture, cupping, and manual therapy were not evaluated independently. Fifth, incorporating objective outcome measures, such as walking distance, gait analysis, imaging findings, or neurophysiological assessments, may provide a more comprehensive evaluation of treatment effects in future trials.

Additionally, baseline imbalances were observed in LANSS scores and comorbidity distribution between groups. The experimental group had a higher baseline LANSS score, indicating greater neuropathic pain severity at baseline, which could bias the estimated treatment effect toward the null (i.e., potentially underestimating the benefit of CIMS). Conversely, the experimental group included a small proportion of participants without reported medical history, which might bias functional outcomes in favor of the experimental group. Although the absolute differences were modest, these imbalances may have influenced the magnitude of observed effects and should be considered when interpreting the results. Although these differences may have acted as potential confounders, the experimental group—despite greater baseline neuropathic pain severity—demonstrated superior improvements in pain and disability outcomes. Nonetheless, the possibility of residual confounding cannot be excluded, and these baseline differences should be carefully considered when interpreting the magnitude of the observed treatment effects. Finally, as this study was conducted within the Korean healthcare system and incorporated elements of traditional Korean medicine, the findings may be most directly applicable to Korean patients.

Future studies should explore the relative contributions of each modality and incorporate longer-term follow-up to better assess the durability of treatment effects and long-term changes in quality of life.

## 5. Conclusions

In patients with DLSS, the addition of CIMS to standard care, including SNRB and pharmacotherapy, resulted in greater reductions in pain and improvements in functional outcomes over 12 weeks compared with standard care alone, extending the findings of our earlier pilot study. These results support the role of CIMS as an effective adjunctive intervention in the non-surgical management of DLSS. The strengths of this study include a randomized controlled design, assessor blinding, and a standardized multimodal intervention targeting both pain and function in patients with neuropathic DLSS. Based on these findings, CIMS has been more actively incorporated into our clinical practice, particularly for patients with persistent radiating pain, and may be applicable to other healthcare settings emphasizing conservative management.

## Figures and Tables

**Table 1:** Inclusion and exclusion criteria.

Inclusion Criteria	
1	Patients < 80 years of age diagnosed with DLSS by clinical symptoms and magnetic resonance imaging (MRI).
2	Patients presenting with LLRP as the primary symptom (LANSS > 7).
3	Patients reporting pain intensity requiring SNRB (VAS > 5).
5	Subjects who voluntarily consented to written consent.
Exclusion Criteria	
1	Patients younger than 20 or older than 80 years.
2	Pregnant patients.
3	Patients with secondary gain (e.g., industrial accident, automobile insurance).
4	Patients with serious systemic comorbidities.
5	Patients with contraindications to the study medications.
6	Patients participating in an interventional study during the study period.
7	Patients with cancer-related pain due to primary or metastatic spinal tumors.
8	Patients unable to complete questionnaires due to communication or cognition.
9	Patients with severe needle phobia preventing participation in acupuncture.

DLSS—degenerative lumbar spinal stenosis; LLRP—lower leg radiating pain; LANSS—Leeds Assessment of Neuropathic Symptoms and Signs; SNRB—selective nerve root block; VAS—visual analog scale.

**Table 2:** Summary of means, standard deviations (SD), and within-/between-group differences.

Group	Value	Baseline	12 Weeks	Difference (Within-Group)	Difference (Between-Group)
Experimental	Mean	4.38	3.21	1.17	Mean
	SD	1.21	1.03	1.03	0.75
	*n*	15	15		0.76
Control	Mean	5.27	4.85	0.42	SD
	SD	1.48	1.26	1.26	1.15
	*n*	15	15		1.15

Note. Within-group difference = baseline − 12 weeks; between-group difference = experimental within-group mean − control within-group mean.

**Table 3:** Parameters for sample size estimation.

*α*/2	0.025
*β*	0.200
Expected difference (*μc* − *μt*)	0.76
Standard deviation (σ)	1.150
Calculated sample size per group (*n*)	35.942
Drop-out rate	0.100
Adjusted sample size per group (*n*_adj)	39.936

Note. Formula for calculating sample size (two independent means, equal variance). *n* = 2 · (z*α*/2 + z*β*)^2^… σ^2^/(*μc* − *μt*)^2^.

**Table 4:** Epidemiological characteristics.

Variable		Experimental Group (*n* = 38)	Control Group (*n* = 39)	*p*-Value
Age (years)		69.74 ± 6.29	69.28 ± 7.32	0.771
BMI (kg/m^2^)		25.03 ± 3.86	24.07 ± 2.61	0.205
LANSS		14.45 ± 1.22	13.72 ± 1.73	0.036
Sex	Male	11 (28.9)	14 (35.9)	0.515
	Female	27 (71.1)	25 (64.1)	
Medical History	No	4 (10.5)	0 (0)	0.037
	Yes	34 (89.5)	39 (100)	
Detailed Medical History	Dermatologic	2	0	
	Ophthalmologic	8	10	
	Otolaryngologic	3	2	
	Gastrointestinal	7	10	
	Endocrine	20	14	
	Neuropsychiatric	7	7	
	Cardiovascular	25	30	
	Respiratory	5	7	
	Hematologic	2	2	
	Urologic	5	8	

Note. Values were presented as the mean ± standard deviation or frequency (%). BMI—body mass index; LANSS—Leeds Assessment of Neuropathic Symptoms and Signs.

**Table 5:** Changes over time in VAS, RMDQ, and ODI by group.

Variable	Group		Visit				*p*-Value *			
		Baseline	Week 4	Week 8	Week 12	Cohen’s d		V	G	V × G
VAS	CIMS	4.73 ± 1.67	3.74 ± 1.68	3.93 ± 2.03	3.98 ± 1.98	−0.55		0.125	0.067	0.040
	Control	4.70 ± 1.95	4.66 ± 1.60	4.79 ± 1.55	4.98 ± 1.68					
RMDQ	CIMS	10.2 ± 4.8	8.47 ± 5.12	9.00 ± 5.58	7.87 ± 5.67	-		0.021	0.712	0.418
	Control	9.77 ± 5.61	9.03 ± 5.62	9.32 ± 4.35	9.13 ± 4.22					
ODI	CIMS	41.48 ± 13.12	36.81 ± 15.07	37.78 ± 14.14	35.19 ± 16.06	−0.41		0.508	0.359	0.010
	Control	39.00 ± 18.68	41.72 ± 16.83	42.51 ± 15.95	41.51 ± 15.37					

All values were presented by mean *±* standard deviation; *: *p*-value calculated by two-factor repeated-measure analysis; Cohen’s d represents the standardized mean difference between groups at week 12. CIMS: comprehensive integrative medical services; V: visit; VAS: visual analog scale; RMDQ: Roland–Morris Disability Questionnaires; ODI: Oswestry Disability Index; G: group; week 4: 29 ± 5 days; week 8: 57 *±* 5 days; week 12: 85 *±* 5 days.

**Table 6:** Changes over time in SF-36 domains by group.

Variable	Group	Visit				*p*-Value *		
		Baseline	Week 4	Week 8	Week 12	V	G	V × G
PCS	CIM	29.85 ± 21.46	40.56 ± 20.08	37.92 ± 21.87	40.85 ± 21.90	0.000	0.604	0.092
	Control	32.76 ± 17.70	35.99 ± 17.24	35.06 ± 19.07	35.79 ± 18.73			
MCS	CIM	48.15 ± 25.29	50.74 ± 24.89	50.29 ± 24.62	54.55 ± 24.77	0.089	0.571	0.685
	Control	44.34 ± 19.24	48.20 ± 25.54	49.95 ± 22.41	48.99 ± 20.52			
PF	CIM	37.00 ± 24.09	46.50 ± 21.70	46.83 ± 22.72	48.67 ± 24.53	0.003	0.685	0.114
	Control	40.16 ± 26.57	44.68 ± 24.66	43.87 ± 25.49	41.13 ± 24.31			
RPH	CIM	22.50 ± 37.91	40.83 ± 41.77	35.00 ± 40.79	40.83 ± 43.29	0.013	0.182	0.743
	Control	18.55 ± 26.59	26.61 ± 32.87	25.00 ± 35.36	29.03 ± 34.82			
BP	CIM	36.92 ± 24.15	47.75 ± 20.91	45.33 ± 20.86	49.25 ± 22.50	0.038	0.726	0.193
	Control	44.92 ± 23.34	45.24 ± 21.83	48.15 ± 22.25	47.50 ± 22.56			
GH	CIM	23.00 ± 16.74	27.17 ± 20.41	24.50 ± 18.02	24.67 ± 19.74	0.322	0.789	0.473
	Control	27.42 ± 17.22	27.42 ± 16.48	23.23 ± 16.00	25.48 ± 16.75			
REP	CIM	43.33 ± 45.61	42.22 ± 42.82	44.44 ± 45.77	52.22 ± 46.88	0.121	0.420	0.328
	Control	25.81 ± 34.11	41.94 ± 46.32	43.01 ± 44.05	41.94 ± 43.01			
EF	CIM	42.83 ± 18.04	43.33 ± 22.22	41.67 ± 19.75	44.17 ± 18.34	0.735	0.816	0.772
	Control	43.39 ± 16.85	39.52 ± 20.06	42.10 ± 17.74	43.39 ± 17.24			
SF	CIM	54.58 ± 28.90	65.42 ± 29.30	60.00 ± 28.50	65.00 ± 29.07	0.173	0.518	0.339
	Control	56.05 ± 29.91	58.06 ± 31.05	57.66 ± 29.35	56.45 ± 27.17			
EWB	CIM	51.87 ± 23.00	52.00 ± 20.98	55.07 ± 22.20	56.80 ± 19.97	0.236	0.961	0.786
	Control	52.13 ± 19.58	53.29 ± 24.60	57.03 ± 21.32	54.19 ± 18.06			

All values were presented by mean ± standard deviation; *: *p*-value calculated by two-factor repeated-measure analysis; CIMS: comprehensive integrative medical services; V: visit; VAS: visual analog scale; G: group; week 4: 29 ± 5 days; week 8: 57 ± 5 days; week 12: 85 ± 5 days; PCS: physical component score; MCS: mental component score; PF: physical functioning; RPH: role limitations due to physical health; BP: body pain; GH: general health; REP: role limitations due to emotional problems; EF: energy/fatigue; SF: social functioning; EWB: emotional well-being.

## Data Availability

The data sets used and/or analyzed during the current study are available from the corresponding author on reasonable request.

## Supplementary Materials

The following supporting information can be downloaded at: https://www.mdpi.com/article/10.3390/medicina62010225/s1. CONSORT 2025 checklist (File S1), CONSORT 2025 flow diagram (File S2), Reference [10] is cited in the Supplementary Materials.

## Abbreviations

The following abbreviations are used in this manuscript:

AE	Adverse event
CIMS	Comprehensive and Integrative Medical Services
DLSS	Degenerative lumbar spinal stenosis
LANSS	Leeds Assessment of Neuropathic Symptoms and Signs
LBP	Low back pain
LLRP	Lower leg radiating pain
MCS	Mental component score
MRI	Magnetic resonance imaging
NIC	Neurogenic intermittent claudication
ODI	Oswestry Disability Index
PCS	Physical component score
RMDQ	Roland–Morris Disability Questionnaire
SNRB	Selective nerve root block
SF-36	36-Item Short-Form Health Survey
VAS	Visual analog scale
